# Author Correction: An interpretable machine learning system for colorectal cancer diagnosis from pathology slides

**DOI:** 10.1038/s41698-024-00581-2

**Published:** 2024-04-03

**Authors:** Pedro C. Neto, Diana Montezuma, Sara P. Oliveira, Domingos Oliveira, João Fraga, Ana Monteiro, João Monteiro, Liliana Ribeiro, Sofia Gonçalves, Stefan Reinhard, Inti Zlobec, Isabel M. Pinto, Jaime S. Cardoso

**Affiliations:** 1 Institute for Systems and Computer Engineering, Technology and Science (INESC TEC), R. Dr. Roberto Frias, Porto, 4200-465 Porto Portugal; 2 Faculty of Engineering, University of Porto (FEUP), R. Dr. Roberto Frias, Porto, 4200-465 Porto Portugal; 3 IMP Diagnostics, Praça do Bom Sucesso, 61, sala 808, Porto, 4150-146 Porto Portugal; 4 Cancer Biology and Epigenetics Group, Research Center of IPO Porto (CI-IPOP) / RISE@CI-IPOP (Health Research Network), Portuguese Oncology Institute of Porto (IPO Porto) / Porto Comprehensive Cancer Center (Porto.CCC), R. Dr. António Bernardino de Almeida 865, Porto, 4200-072 Porto Portugal; 5 Doctoral Programme in Medical Sciences, School of Medicine and Biomedical Sciences - University of Porto (ICBAS-UP), R. Jorge de Viterbo Ferreira 228, Porto, 4050-313 Porto Portugal; 6 Department of Pathology, IPO-Porto, R. Dr. António Bernardino de Almeida 865, Porto, 4200-072 Porto Portugal; 7 Institute of Pathology, University of Bern, Uni Bern, Murtenstrasse 31, Bern, 3008 Bern Switzerland

**Keywords:** Cancer imaging, Colorectal cancer, Colorectal cancer, Colorectal cancer

Correction to: *npj Precision Oncology* 10.1038/s41698-024-00539-4, published online 05 March 2024

In the original version of this article, the wrong figure files were inadvertently supplied for Figs. 9 and 11. The original article has been corrected.

