# Supplementary material for: An interpretable machine learning system for colorectal cancer diagnosis from pathology slides
Source: NPJ Precis Oncol. 2024 Mar 5;8:56. doi: 10.1038/s41698-024-00539-4 (PMC10914836; doi:10.1038/s41698-024-00539-4)
Supplement: Supplementary file 1 — Reporting Summary [file 41698_2024_539_MOESM1_ESM.pdf]

nature portfolio

Corresponding author(s): Pedro C. Neto

Last updated by author(s): Jan 16, 2024

Reporting Summary

Nature Portfolio wishes to improve the reproducibility of the work that we publish. This form provides structure for consistency and transparency in reporting. For further information on Nature Portfolio policies, see our Editorial Policies and the Editorial Policy Checklist.

Statistics

For all statistical analyses, confirm that the following items are present in the figure legend, table legend, main text, or Methods section.

n/a	Confirmed	
☐	☒	The exact sample size (n) for each experimental group/condition, given as a discrete number and unit of measurement
☐	☒	A statement on whether measurements were taken from distinct samples or whether the same sample was measured repeatedly
☐	☒	The statistical test(s) used AND whether they are one- or two-sided<br>*Only common tests should be described solely by name; describe more complex techniques in the Methods section.*
☐	☒	A description of all covariates tested
☐	☒	A description of any assumptions or corrections, such as tests of normality and adjustment for multiple comparisons
☐	☒	A full description of the statistical parameters including central tendency (e.g. means) or other basic estimates (e.g. regression coefficient) AND variation (e.g. standard deviation) or associated estimates of uncertainty (e.g. confidence intervals)
☐	☒	For null hypothesis testing, the test statistic (e.g. F, t, r) with confidence intervals, effect sizes, degrees of freedom and P value noted<br>*Give P values as exact values whenever suitable.*
☒	☐	For Bayesian analysis, information on the choice of priors and Markov chain Monte Carlo settings
☒	☐	For hierarchical and complex designs, identification of the appropriate level for tests and full reporting of outcomes
☒	☐	Estimates of effect sizes (e.g. Cohen's d, Pearson's r), indicating how they were calculated

Our web collection on statistics for biologists contains articles on many of the points above.

Software and code

Policy information about availability of computer code

Data collection	There was no specific code for data collection.
Data analysis	For data analysis Pytorch, numpy and similar deep learning and scientific computing libraries have been used.

For manuscripts utilizing custom algorithms or software that are central to the research but not yet described in published literature, software must be made available to editors and reviewers. We strongly encourage code deposition in a community repository (e.g. GitHub). See the Nature Portfolio guidelines for submitting code & software for further information.

Data

Policy information about availability of data

All manuscripts must include a data availability statement. This statement should provide the following information, where applicable:

- Accession codes, unique identifiers, or web links for publicly available datasets
- A description of any restrictions on data availability
- For clinical datasets or third party data, please ensure that the statement adheres to our policy

This data composed of WSI and respective labels has been released under CC BY-NC. This release is part of the efforts of IMP Diagnostics and INESC TEC to advance science and share knowledge. It can be found in the following public repository: https://doi.org/10.25747/fb1q-j507

Research involving human participants, their data, or biological material

Policy information about studies with human participants or human data. See also policy information about sex, gender (identity/presentation), and sexual orientation and race, ethnicity and racism.

Reporting on sex and gender	There are no sex/gender differences between colorectal histology samples, this is, we cannot distinguish men/women cases based on colorectal histology alone and, as such, ensuring equal representation of each or disaggregation of the results by sex/gender might not be relevant for this particular work. Also, we gathered our data retrospectively from our Department's archive, sequentially selecting all cases that matched the study's diagnostic categories (consecutive sampling). Thus, our sampling represents the prevalence across gender/sex of histology samples of the study population. We have added this information in our article.
Reporting on race, ethnicity, or other socially relevant groupings	Such as for sex and gender, we cannot distinguish races/ ethnicities based on colorectal histology images. Also, race/ethnicity data were not available for these patients. As previously explained, as we gathered our data retrospectively from our Department's archive, sequentially selecting all cases that matched the study's diagnostic categories (consecutive sampling) our sampling represents the prevalence across different races/ethnicities of the study population.
Population characteristics	As previously described, we followed a consecutive sampling strategy to retrieve the cases from our archive, thus the distribution in our dataset across samples is a reflection of the distribution of colorectal samples of the study's population. We have only collected the case digitized image and corresponding diagnosis for this study (no personal data was retrieved for this study).
Recruitment	We followed a consecutive sampling strategy to retrieve the cases from our archive (sequentially selecting all cases that matched the study's diagnostic categories), thus the distribution in our dataset across samples is representative of the distribution of colorectal samples of the study's population.
Ethics oversight	All procedures performed in studies involving human participants were in accordance with national law and with the 1964 Helsinki Declaration and its later amendments or comparable ethical standards. Data collection and usage was performed in accordance with national legal and ethical standards applicable to this type of data.. Ethical review and approval were waived for this study due to usage of samples exclusively for research purposes in retrospective studies and the fact that the samples were anonymized.

Note that full information on the approval of the study protocol must also be provided in the manuscript.

Field-specific reporting

Please select the one below that is the best fit for your research. If you are not sure, read the appropriate sections before making your selection.

☒ Life sciences ☐ Behavioural & social sciences ☐ Ecological, evolutionary & environmental sciences

For a reference copy of the document with all sections, see nature.com/documents/nr-reporting-summary-flat.pdf

Life sciences study design

All studies must disclose on these points even when the disclosure is negative.

Sample size	Deep learning solutions are known for requiring large amounts of data. We introduce a sample size that is significantly larger than the sample size of other works in the literature that leverage deep learning.
Data exclusions	We followed a consecutive sampling strategy to retrieve the cases from our archive (sequentially selecting all cases that matched the study's diagnostic categories); Exclusion criteria were: other diagnostic categories that did not fit the study objectives and images which had low quality digitization/artifacts, precluding assessment.
Replication	The experiments were run after following all the deterministic setup of the deep learning library used. As such, on a similar machine it is possible to fully replicate the results, as we did to confirm and to test the proposed "sampling" approach.
Randomization	In our study the sampling was consecutive. Even though it was not randomly chosen, it is a representative sample of the study population. Regarding allocation into the different testing groups.
Blinding	All cases were firstly labeled/diagnosed blinded to the initial diagnostic report. This assessment was then compared with the initial diagnosis and, if they matched, no further steps were taken; if diagnosis differed, another pathologist was consulted to serve as tie-breaker.

Reporting for specific materials, systems and methods

We require information from authors about some types of materials, experimental systems and methods used in many studies. Here, indicate whether each material, system or method listed is relevant to your study. If you are not sure if a list item applies to your research, read the appropriate section before selecting a response.

Materials & experimental systems

n/a	Involved in the study
☒	☐ Antibodies
☒	☐ Eukaryotic cell lines
☒	☐ Palaeontology and archaeology
☒	☐ Animals and other organisms
☒	☐ Clinical data
☒	☐ Dual use research of concern
☒	☐ Plants

Methods

n/a	Involved in the study
☒	☐ ChIP-seq
☒	☐ Flow cytometry
☒	☐ MRI-based neuroimaging

Plants

Seed stocks	*Report on the source of all seed stocks or other plant material used. If applicable, state the seed stock centre and catalogue number. If plant specimens were collected from the field, describe the collection location, date and sampling procedures.*
Novel plant genotypes	*Describe the methods by which all novel plant genotypes were produced. This includes those generated by transgenic approaches, gene editing, chemical/radiation-based mutagenesis and hybridization. For transgenic lines, describe the transformation method, the number of independent lines analyzed and the generation upon which experiments were performed. For gene-edited lines, describe the editor used, the endogenous sequence targeted for editing, the targeting guide RNA sequence (if applicable) and how the editor was applied.*
Authentication	*Describe any authentication procedures for each seed stock used or novel genotype generated. Describe any experiments used to assess the effect of a mutation and, where applicable, how potential secondary effects (e.g. second site T-DNA insertions, mosiacism, off-target gene editing) were examined.*
