# Supplementary material for: An interpretable machine learning system for colorectal cancer diagnosis from pathology slides
Source: NPJ Precis Oncol. 2024 Mar 5;8:56. doi: 10.1038/s41698-024-00539-4 (PMC10914836; doi:10.1038/s41698-024-00539-4)
Supplement: Supplementary file 2 — Supplementary Material [file 41698_2024_539_MOESM2_ESM.pdf]

Supplementary material for:
An interpretable machine learning system for colorectal cancer diagnosis from pathology slides

Pedro C. Neto[a,b,1], Diana Montezuma[c,f,d,1], Sara P. Oliveira[a,b,1], Domingos Oliveira[c], João Fraga[e], Ana Monteiro[c], João Monteiro[c], Liliana Ribeiro[c], Sofia Gonçalves[c], Stefan Reinhard[g], Inti Zlobec[g], Isabel M. Pinto[c], Jaime S. Cardoso[a,b]

[a] *Institute for Systems and Computer Engineering, Technology and Science (INESC TEC), R. Dr. Roberto Frias, Porto, 4200-465, Porto, Portugal*
[b] *Faculty of Engineering, University of Porto (FEUP), R. Dr. Roberto Frias, Porto, 4200-465, Porto, Portugal*
[c] *IMP Diagnostics, Praça do Bom Sucesso, 61, sala 809, Porto, 4150-146, Porto, Portugal*
[d] *Cancer Biology and Epigenetics Group, IPO-Porto, R. Dr. António Bernardino de Almeida 865, Porto, 4200-072, Porto, Portugal*
[e] *Department of Pathology, IPO-Porto, R. Dr. António Bernardino de Almeida 865, Porto, 4200-072, Porto, Portugal*
[f] *School of Medicine and Biomedical Sciences, University of Porto (ICBAS), R. Jorge de Viterbo Ferreira 228, Porto, 4050-313, Porto, Portugal*
[g] *Institute of Pathology, University of Bern, Uni Bern, Murtenstrasse 31, Bern, 3008, Bern, Switzerland*

Keywords: Clinical Prototype, Colorectal Cancer, Interpretable Artificial Intelligence, Deep Learning, Whole-Slide Images

Supplementary Results

Supplementary Tables 1, 2 and 3 show a consistent advantage of the proposed model when trained on the CRS10K dataset over the other models. This metrics provide more insights on the performance of the model and corroborate the development of the CRS10K model.

On Supplementary Table 2 it is visible that despite having the same accuracy, iMIL4Path provides less separable confidences when compared with our method trained on CRS10K. This is to be expected attending to the distribution of the confidences on the Figure 10 of the main document.

Since this metrics have been computed in a binary setting (cancer vs non-cancer) they relate to the binary accuracy presented in the main document.

Supplementary Table 1. Additional metrics measured on the Test set for the main methods evaluated in this document. We present the area under the precision recall (PR-AUC) curve, the area under the receiver operating characteristic (ROC-AUC) curve, and the F1-score.

**Method**	**PR-AUC**	**F1-Score**	**ROC-AUC**
iMIL4Path	0.996	0.969	0.984
**Ours (CRS4K)**	0.994	0.960	0.978
**Ours (CRS10K) wo/ Agg**	**0.998**	**0.977**	**0.992**

Supplementary Figure 1 shows the precision-recall curves for the three models evaluated on the prototype test set. Once again the F1-Score also highlights the performance of the proposed model to mitigate both errors.

Evaluation of the confidence of the model in the PAIP dataset shows that in two of the three approaches, the number of incorrect samples is one or zero, as such, there is no density estimation for wrong samples in their confidence plot as seen in Supplementary Figure 2. Yet, it is visible the shift towards higher values of confidence in the proposed approach trained on the CRS10K when compared to the method of iMIL4Path. The version trained on CRS4K shows very little separability between the confidence of correct and incorrect predictions.

[1] These authors contributed equally.

Supplementary Table 2. Additional metrics measured on the Prototype set for the main methods evaluated in this document. We present the area under the precision recall (PR-AUC) curve, the area under the receiver operating characteristic (ROC-AUC) curve, and the F1-score. The best values per column are in bold.

Method	PR-AUC	F1-Score	ROC-AUC
iMIL4Path	0.993	0.959	0.979
Ours (CRS4K)	0.966	0.926	0.926
Ours (CRS10K) wo/ Agg	**0.999**	**0.980**	**0.996**

Supplementary Table 3. Additional metrics measured on joint set of all the test datasets for the main methods evaluated in this document. We present the area under the precision recall (PR-AUC) curve, the area under the receiver operating characteristic (ROC-AUC) curve, and the F1-score. The best values per column are in bold.

Method	PR-AUC	F1-Score	ROC-AUC
iMIL4Path	0.993	0.943	0.965
Ours (CRS4K)	0.993	0.965	0.968
Ours (CRS10K) wo/ Agg	**0.998**	**0.982**	**0.991**

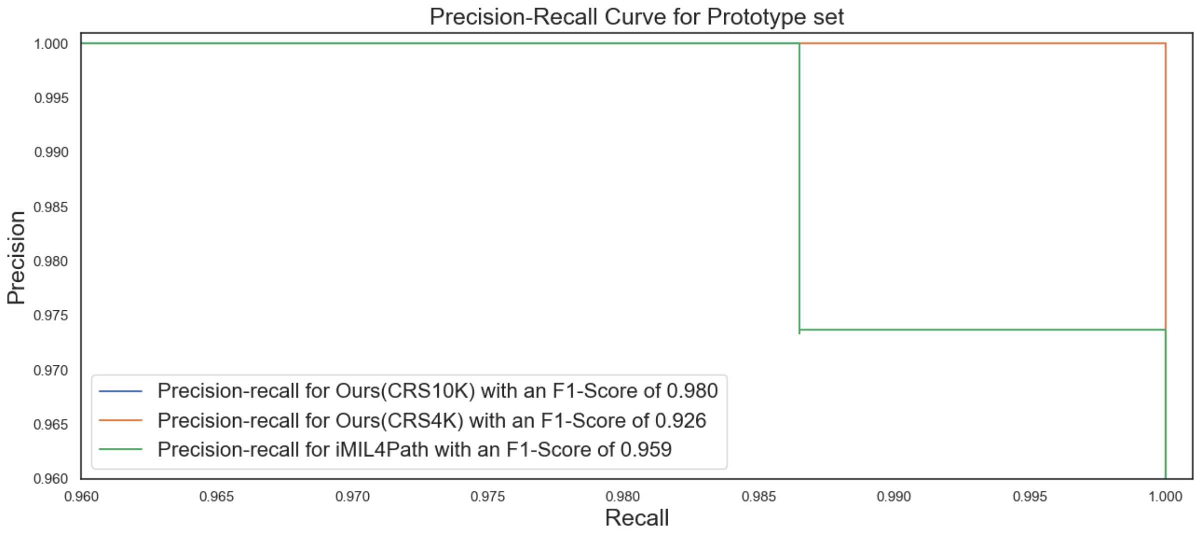

Supplementary Figure 1. **Precision-recall curve on the on the Prototype set:** For the three distinct models, we have calculated the Precision-recall curve on this dataset. Includes an indication of the F1-Score for each of the different models. The blue line represents the curve of Our method when trained on CRS10K, while the orange line shows the same method when trained on CRS4K. The green line is the curve of iMIL4Path.

Inspecting the predictions' confidence for the three models, for the TCGA dataset, indicates a behaviour in line with the accuracy-based performance (Supplementary Figure 3). Moreover, a confidence shift of wrong predictions' confidence towards smaller values is clearly visible in the plot corresponding to the model trained on CRS10K. The shown gap of 0.2 between the confidence of correct and wrong predictions, indicates that it is possible to quantify the uncertainty of the model and avoid the majority of the wrong predictions. In other words, when the uncertainty is above a learnt threshold, then the model refuses to make any prediction. It is extremely useful in models designed as a second opinion system.

On the prototype set (Supplementary Figure 4), the gains are less evident, which can be assumed to be related to the lower data quantity. However, performances above 91% are achieved by all the three models, with our model trained on the CRS10K data being the one that requires a larger rejection rate to achieve such value. Nonetheless, as the rejection rate increases the performance of that model improves to 100% accuracy at less than 50% rejection.

On the TCGA set, the performance is already significantly different without any rejection. The difference between

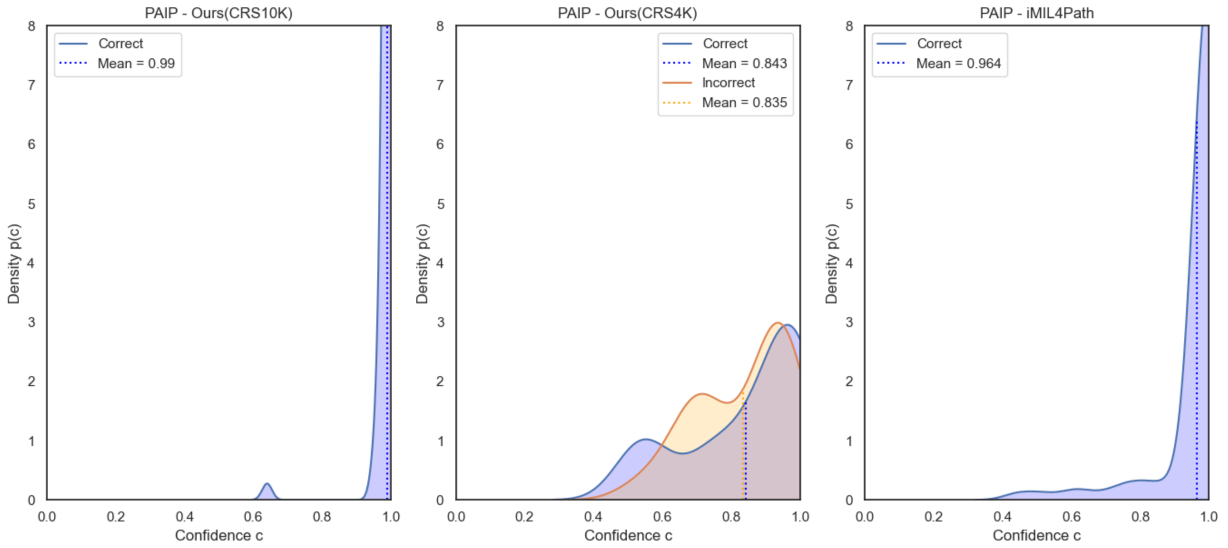

Supplementary Figure 2. **Confidence analysis for correct and incorrect predictions on the PAIP dataset:** Kernel density estimation of the confidences of correct and incorrect predictions performed on the three-class classification problem by three distinct models on the PAIP dataset. The plots represent, from left to right, the proposed method trained on CRS10K, the proposed method trained on CRS4K and iMIL4Path. In each plot, the blue line defines the density function of the correct samples and the blue dashed line the mean confidence of those samples. On the other hand, the orange solid and dashed lines represent the same for incorrect predictions.

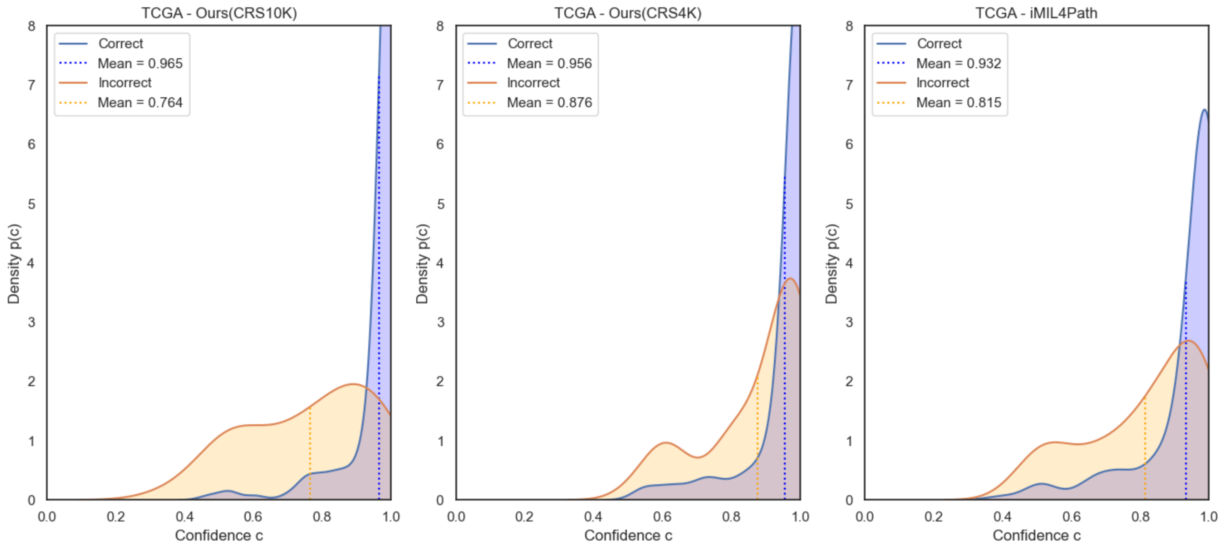

Supplementary Figure 3. **Confidence analysis for correct and incorrect predictions on the TCGA dataset:** Kernel density estimation of the confidences of correct and incorrect predictions performed on the three-class classification problem by three distinct models on the TCGA dataset. The plots represent, from left to right, the proposed method trained on CRS10K, the proposed method trained on CRS4K and iMIL4Path. In each plot, the blue line defines the density function of the correct samples and the blue dashed line the mean confidence of those samples. On the other hand, the orange solid and dashed lines represent the same for incorrect predictions.

the performance of the best performing model and the remaining is kept at the different rates. At 16% rejection rate, as seen in Supplementary Figure 5, the accuracy of the model trained on the CRS10K dataset is 91.54%. At 50% the performance becomes 97.26%.

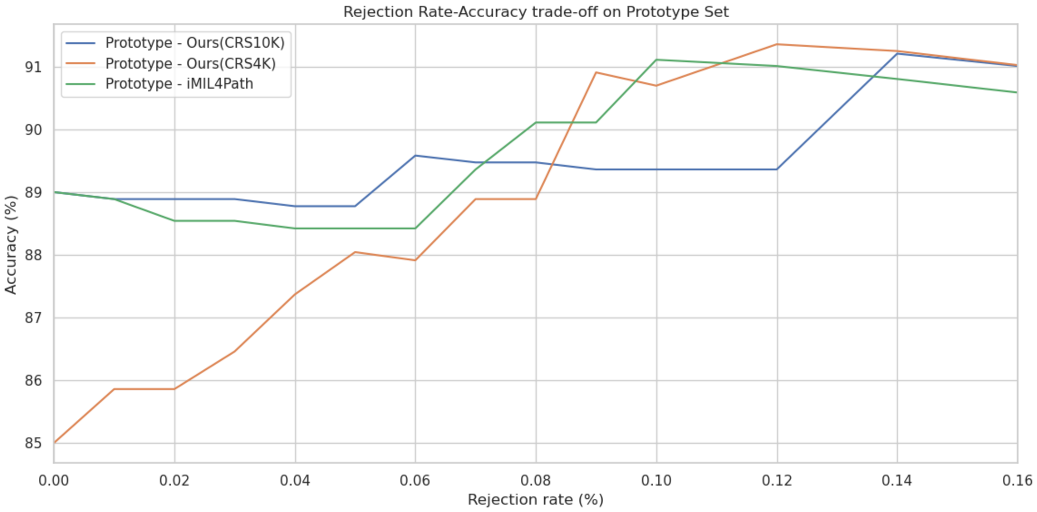

Supplementary Figure 4. **Accuracy-vs-Rejection-rate for the models evaluated on the Prototype set**. Relation between the accuracy and the percentage of samples not classified by the model. Both axes are in percentage. The blue line represents Our method when trained on CRS10K, while the orange line shows the same method when trained on CRS4K. The green line is for iMIL4Path.

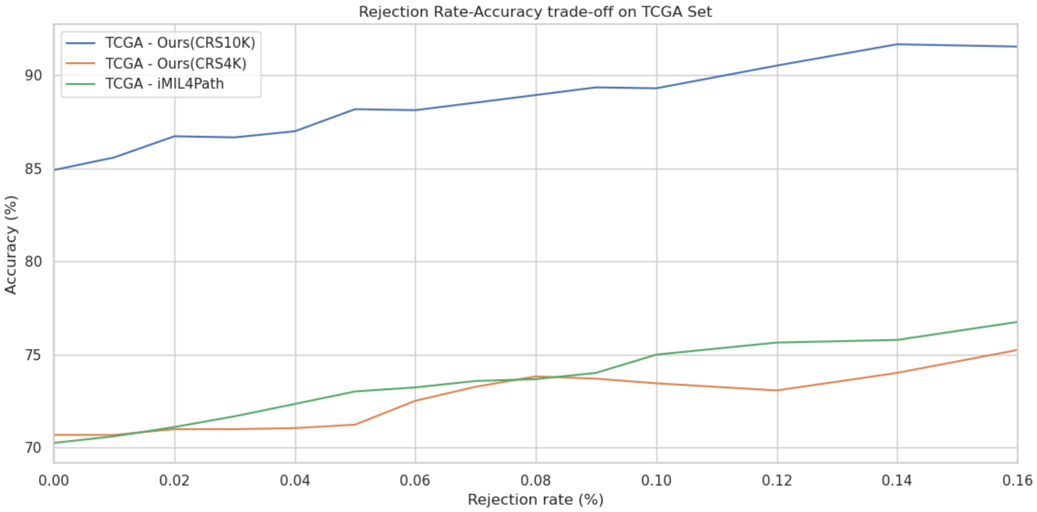

Supplementary Figure 5. **Accuracy-vs-Rejection-rate for the models evaluated on the TCGA dataset**. Relation between the accuracy and the percentage of samples not classified by the model. Both axes are in percentage. The blue line represents Our method when trained on CRS10K, while the orange line shows the same method when trained on CRS4K. The green line is for iMIL4Path
